# Pattern of Local Recurrence and Distant Metastasis in Breast Cancer By Molecular Subtype

**DOI:** 10.7759/cureus.924

**Published:** 2016-12-09

**Authors:** Xingrao Wu, Ayesha Baig, Goulnar Kasymjanova, Kamran Kafi, Christina Holcroft, Hind Mekouar, Annie Carbonneau, Boris Bahoric, Khalil Sultanem, Thierry Muanza

**Affiliations:** 1 Department of Radiation Oncology, KunMing Medical University Yunnan Provincial Cancer Hospital, Kunming, People’s Republic of China; 2 Department of Oncology, Division of Radiation Oncology, Segal Cancer Center-Jewish General Hospital, McGill University, Montréal, Canada; 3 Department of Medicine and Pulmonary Oncology, Jewish General Hospital, McGill University, Montréal, Canada; 4 Statistical Consultation Service Centre for Clinical Epidemiology, Jewish General Hospital, McGill University, Montréal, Canada

**Keywords:** breast cancer, molecular subtype, pattern, recurrence

## Abstract

Background and Purpose: No longer considered a single disease entity, breast cancer is being classified into several distinct molecular subtypes based on gene expression profiling. These subtypes appear to carry prognostic implications and have the potential to be incorporated into treatment decisions. In this study, we evaluated patterns of local recurrence (LR), distant metastasis (DM), and association of survival with molecular subtype in breast cancer patients in the post–adjuvant radiotherapy setting.

Material and Methods: The medical records of 1,088 consecutive, non-metastatic breast cancer patients treated at a single institution between 2004 and 2012 were reviewed. Estrogen/progesterone receptors (ER/PR) and human epidermal growth factor receptor-2 (HER2) enrichment were evaluated by immunohistochemistry. Patients were categorized into one of four subtypes: luminal-A (LA; ER/PR+, HER2-, Grade 1-2), luminal-B (LB; ER/PR+, HER2-, Grade > 2), HER2 over-expression (HER2; ER/PR-, HER2+), and triple negative (TN; ER/PR-, HER2-).

Results: The median follow-up time was 6.9 years. During the follow-up, 16% (174/1,088) of patients failed initial treatment and developed either LR (48) or DM (126). The prevalence of LR was the highest in TN (12%) and the lowest in LA (2%). Breast or chest wall relapse was the most frequent site (≈80%) of recurrence in LA, LB, and HER2 subtypes, whereas the regional lymph nodes and chest wall were the common sites of relapse in the TN group (50.0%). DM rates were 6.4% in LA, 12.1% in LB, 19.2% in HER2, and 27.4% in TN subgroups. Five-year survival rates were 84%, 83%, 84%, and 77% in the LA, LB, HER2 and TN subgroups, respectively. There was a statistically significant association between survival and molecular subtypes in an univariate analysis. In the adjusted multivariate analysis, the following variables were independent prognostic factors for survival: T stage, N stage, and molecular subtype.

Conclusions: Of the four subtypes, the LA subtype tends to have the best prognosis, fairly high survival, and low recurrent or metastases rates. The TN and HER2 subtypes of breast cancer were associated with significantly poorer overall survival and prone to earlier recurrence and metastases. Our results demonstrate a significant association between molecular subtype and survival. The risk of death and relapse/metastases increases fewfold in TN compared to LA. Future prospective studies are warranted and could ultimately lead to the tailoring of adjuvant radiotherapy treatment fields based on both molecular subtype and the more conventional clinicopathologic characteristics.

## Introduction

Breast cancer (BC) is a heterogeneous disease with widely varying clinical behaviors and treatment responses, despite similarities in standard clinicopathologic characteristics, such as the histological type, tumour size, lymph node status, lymphovascular space invasion, and grade. This diversity in natural history may reflect the underlying molecular biology of the disease. Four major molecular subtypes of BC have been elucidated through gene expression profiling and include luminal-A (LA), luminal-B (LB), human epidermal growth factor receptor-2-enriched (HER2), and basal subtypes [1-3]. Because gene expression profiling is resource intensive and not currently feasible for routine use, BC molecular subtypes can be approximated by standard immunohistochemical features with the LA subtype representing hormone receptor-positive tumours with low proliferative activity, the LB subtype representing hormone receptor-positive tumours with high proliferative activity, the HER2 subtype representing HER2+ tumours, and the basal subtype representing triple negative (TN) disease with no expression of hormone receptors or HER2 [4]. 

Molecular subtypes in BC have been correlated with differences in recurrence rates and survival. Typically, LA subtypes have the most favourable outcomes and TN subtypes experience higher rates of locoregional recurrence and DM, as well as lower survival rates. Controversies with regard to the optimal locoregional management of BC exist, and molecular subtypes are being increasingly considered for prognostication and therapy decisions [1, 4-5]. With the prognostic information garnered from molecular subtype analyses, there is potential to further refine and personalize treatment for BC patients. A key component in tailoring treatment based on molecular subtype is the better understanding of their patterns of local, regional, and distant recurrence.

This study evaluated the pattern of recurrence and disease-free and overall survival by molecular subtype in patients with newly diagnosed BC patients treated with either breast-conserving therapy or mastectomy and adjuvant radiotherapy.  

## Materials and methods

### Patients

The medical records of female histologically-confirmed breast cancer patients treated with radiation therapy at the Jewish General Hospital between 2004 and 2012 were retrospectively reviewed. Stage 4 patients were excluded. All patients underwent nodal staging and received external beam radiotherapy (EBRT) with a dose of 42-50 Gy in 16-25 fractions. In addition, patients aged 60 years or younger and/or patients with positive surgical margins or margins less than 2 mm were prescribed a boost, consisting of an additional 10-15 Gy in four to six fractions, to the tumor bed. Clinicopathologic and treatment information were collected and included age, stage, histology, margin status (< 3 mm vs. ≥ 3 mm), and radiotherapy and systemic treatment details.

The Jewish General Hospital Ethics Review Board approved this retrospective study (approval # CR1366). Informed patient consent was obtained at the time of treatment. 

### Molecular subtype categorization

Molecular subtypes were approximated using hormone receptor status, HER-2 status, and histologic grade. Patients were categorized into four subtype groups: LA (ER/PR+, HER2-, Grade 1-2) LB (ER/PR+, HER2-, Grade > 2); HER2+ (ER/PR+ or ER/PR-, HER-2+); and TN (ER/PR-, HER2-). ER/PR status was determined on the basis of immunohistochemistry (IHC) staining. Tumours were considered HER2+ if they scored 3+ on IHC or if they were 2+ on IHC and demonstrated HER2 amplification on fluorescence in situ hybridization (FISH) [4]. The histologic grade was used as an approximation of the tumour proliferation marker Ki67 [6].

### Follow-up and study end points

Follow-up started on the day of pathology-proven diagnosis and ended on the date the patient was last observed or the date of death. The database was frozen for the statistical analyses on October 2014. Patients who were alive at end of the study or lost to follow-up were censored. One thousand and eighty-eight out of 1,189 patients were included in this analysis. The two primary variables of interest were locoregional recurrence (LRR) and distance metastasis (DM). Patterns of LRR were evaluated and were categorized as local breast/chest wall recurrence and regional lymph node recurrence (axillary, supraclavicular, internal mammary, contralateral). DM was categorized as lung, liver, brain, and bone. Study end points were defined as:

- Overall survival (OS) was defined as the time from the diagnosis to death from any cause

- Progression-free survival (PFS) was defined as the time from the diagnosis to recurrence and/or metastasis, whichever is the earliest. 

- Local recurrence-free survival (LRFS) defined as a time elapse between diagnosis and recurrence or death dates

- Distant metastasis-free survival (DMFS) is defined as a time elapsed between diagnosis and metastasis or death dates. 

### Statistics

The demographic and clinical characteristics of patients in different molecular subtypes were first examined using Pearson c2 tests for categorical variables and ANOVA test for continuous variables. The Kaplan-Meier analysis was used for OS, LRFS, and DMFS with a log-rank test to assess the significance of molecular subtypes for those three outcome variables. The effect of significant clinicodemographic variables on the outcome (OS) was analyzed in univariate Cox regression analysis. The variables assessed in the univariate analysis were age, menopausal status, stage (T&N), tumor grade, histology, chemotherapy, radiation therapy (RT) boost, and molecular subtypes. In the multivariate Cox regression analysis, we adjusted the outcome for the variables that were significant in the univariate analysis: stage (T&N), chemotherapy, grade, and molecular subtypes. A p-value of < 0.05 was considered significant. Hazard ratios and 95% CI were calculated for each variable in the model. All analyses were performed using the Statistical Package for Social Sciences (SPSS) 17.0 (IBM SPSS Statistics, Armonk, NY). 

## Results

A cohort of 1,189 consecutive female patients with breast cancer diagnosed between 2004 and 2012 was identified. In all, 101 patients were excluded from the analysis because of missing molecular subtype information. Therefore, 1,088 patients were available for analyses. The minimum follow-up was 10 months with a median follow-up of 6.9 years. During the follow-up, 80 patients died, eight were lost to follow-up, 48 patients relapsed, and 126 developed distant metastases. All patients received EBRT ± boost RT. For systemic treatment, 63% of patients received cytotoxic chemotherapy, 71.8% received hormonal therapy, and 12.1% of received trastuzumab (Table 1).

**Table 1:** Clinicopathologic Characteristics of Patients 1 - Mixed = lobular+ductal, lobular+DCIS, ductal+DCIS 2 - Others = tubular, medullar, and mucinous 3 - Boost = radiation boost to breast surgical cavity DCIS: ductal carcinoma in situ; DM: distant metastasis; HER2: human epidermal growth factor receptor-2; LA: luminal-A; LB: luminal-B; LR: local recurrence; SD: standard deviation; TN: triple negative

Covariates	Total	%	L A	%	L B	%	HER2	%	TN	%	P value
	N=1088	100	N=644	59	N=173	16	N=125	12	N=146	13	
Age											<0.001
Mean + SD (years)	59.1±12		60.6±12		57.4±13		57.0+2		56.6+13		
Age at diagnosis											0.013
≤ 44	139	12.7	66	10.2	28	16.2	18	14	27	18.5	
> 44	949	87.3	578	89.8	145	83.8	107	86	119	81.5	
Menopausal status											0.005
Pre-menopausal	277	25.5	139	21.6	52	30.0	41	33.0	45	31.0	
Post-menopausal	809	74.5	504	78.4	121	70.0	83	67.0	101	69.0	
T stage											<0.001
T1	625	57.4	432	67.3	69	39.9	61	42.8	61	41.8	
T2	361	33.2	163	25.3	81	46.8	53	42.4	64	43.8	
T3	62	5.7	33	5.1	11	6.4	5	4.0	13	8.9	
T4	40	3.7	14	2.2	12	6.9	6	4.8	8	5.5	
N-stage											0.001
N0	750	68.9	500	77.6	93	53.8	63	50.4	94	64.4	
N1	244	22.4	101	15.7	60	34.7	49	39.2	34	23.3	
N2	83	7.6	38	5.9	18	10.4	13	10.4	14	9.6	
N3	11	1.0	5	0.8	2	1.2	0	0	4	2.7	
TNM Stage											<0.001
I	547	50.3	393	61.0	57	33.0	48	38.4	49	33.5	
II	399	36.7	191	29.7	59	49.0	59	47.2	64	43.9	
III	142	13.0	60	9.3	18	18.0	18	14.4	33	22.6	
Tumor grade											<0.001
1-2	729	47.3	642	100	0	0	54	43.2	32	22.9	
3	355	42.7	40	68.1	173	100	71	56.8	110	77.1	
Histology Type											<0.001
Invasive lobular	129	11.9	102	15.8	13	7.5	7	5.6	7	4.8	
Invasive ductal	890	81.8	490	76.0	154	89.0	111	88.8	135	92.4	
Mixed^1^	40	3.7	30	4.7	4	2.3	5	4.0	1	0.6	
Others^2^	29	2.6	22	3.5	2	1.2	2	1.6	3	2.2	
Surgical											0.152
Lumpectomy	981	90.0	588	91.3	148	85.5	114	91.2	131	89.7	
Mastectomy	107	10.0	56	8.7	25	14.5	11	8.8	15	10.3	
Resection margin											0.735
Negative	1046	96.1	616	95.7	167	96.5	122	97.6	141	96.6	
Positive	42	3.9	28	4.3.	6	3.5	3	2.4	5	3.4	
Margin distance											0.646
≤ 3mm	923	88.8	542	84.2	146	84.4	106	84.8	129	84.8	
> 3mm	165	15.2	102	15.8.	27	15.2	19	15.2	17	15.2	
Chemotherapy											<0.001
Neoadjuvant	108	9.9	44	6.8	17	9.8	19	15.2	28	19.2	
Adjuvant	578	53.1	294	45.7	117	67.6	76	60.8	91	62.3	
None	402	36.9	306	47.5	39	22.5	30	24.0	27	18.5	
Hormonal therapy											<0.001
Yes	781	71.8	554	86.0	155	89.6	69	55.2	3	2.1	
No	307	28.2	90	14.0	18	10.4	56	44.8	143	97.9	
Herceptin therapy											<0.001
Yes	140	12.9	15	2.3	19	89.0	104	83.2	2	1.4	
No	948	87.1	629	97.7	154	11.0	21	16.2	144	98.6	
Radiation (RT)											<0.001
Boost^3^	533	51.0	302	46.9	83	48.0	65	52.0	80	54.8	
No boost	555	49.0	342	53.1	90	52.0	60	48.0	66	45.2	
LR	48	4.4	13	2.0	7	4.0	10	8.0	18	12.0	<0.001
DM	126	11.6	41	6.4	21	12.1	24	19.2	40	27.4	<0.001
Death	80	7.4	25	3.9	11	6.4	12	9.7	32	21.9	<0.001

The prevalence of different molecular subtypes was: LA - 644 (59%), LB - 173 (16%), TN - 146 (13%), and HER2 - 125 (12%). Table 1 presents baseline characteristics of molecular subtypes. In our cohort, TN tumors, when compared to other subtypes, occurred more often in younger women, in a more advanced stage, and more often invasive ductal histology with Grade 3 tumor.

**Figure 1:**
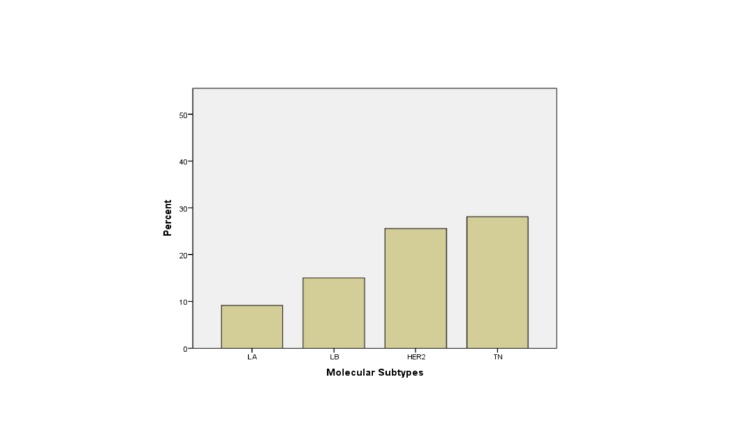
Failure Rate (Local Recurrence + Distal Metastases) Among Different Groups of Molecular Subtypes

Sixteen percent of patients (174/1,088) failed initial treatment and developed either recurrence (48) or distal metastasis (126) (Figure 1).

The failure rate was the highest in TN tumors. Detailed rates of LR and DM are shown in Table 2.

**Table 2:** Recurrence and Distant Metastasis by Molecular Subtypes HER2: human epidermal growth factor receptor-2; HR: hazard ratio; LA: luminal-A; LB: luminal-B; TN: triple negative

Recurrence Pattern	LA%	LB%	HER2%	TN%	P value
	N = 12	N = 6	N = 10	N = 18	
Breast	5 (41.7)	2 (33.3)	5 (50.0)	2 (11.1)	0.451
Chest wall	5 (41.7)	32 (50.0)	3 (30.0)	7 (38.9)	
Ipsilateral lymph nodes	1 (8.3)	1 (16.7)	2 (20.0)	8 (44.4)	
Internal mammary nodes	1 (8.3)	0 (0.0)	0 (0)	1 (5.6)	
Contralateral	0	0 (9.0)	0 (0)	0 (0.0)	
Metastasis pattern	LA%	LB%	HER2%	TN%	P value
	N = 40	N = 21	N = 25	N = 40	
Bone	22 (55.0)	9 (42.9)	9 (36.0)	10 (25.0)	0.379
Lung	11 (27.5)	8 (38.1)	8 (32.0)	17 (42.5)	
Brain	1 (2.5)	3 (14.3)	5 (20.0)	7 (17.5)	
Liver	4 (10.0)	1 (4.8)	2 (8.0)	3 (7.5)	
Others	2 (5.0)	0	1 (4.0)	3 (7.5)	

Of the four subtypes, the highest LR rate was among TN patients - 12% (18/146), followed by HER2 - 8% (10/125), then by LB - 4% (7/173). Luminal A tumors tend to have the lowest LR rate - 2% (13/644). The most common sites of LR were breast and chest wall (32/48), followed by regional lymph nodes (16/48) for LA, LB, and HER2 with breast and chest wall being about 80% of recurrence. However, for TN, the rate of recurrence in regional lymph nodes increased up to 50%.

The prevalence of DM was highest among TN - 27.4% (40/146) as well, followed by HER2 - 19.2% (24/125), then by LB - 12.1% (21/173). LA had the lowest rate of DM - 6.4 % (41/644) (p < 0.001). The most common sites of DM were bone (39%) and lung (35%) for all four subtypes (Table 2). Prevalence of brain, liver, and pelvis metastasis was higher in TN and HER2 groups when compared to LA and LB (Table 2).

**Figure 2:**
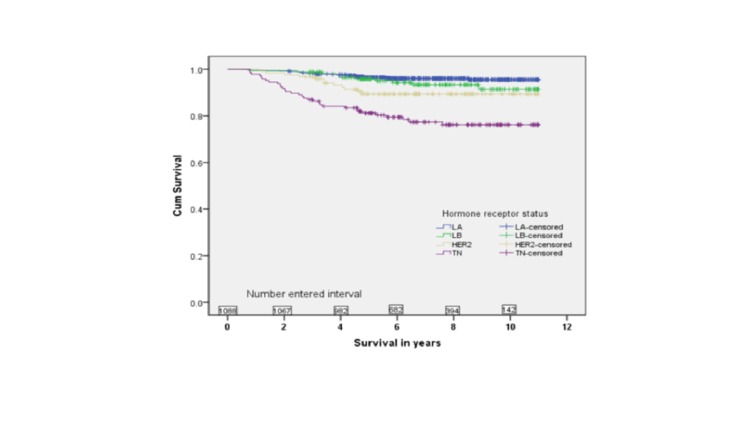
Overall Survival by Molecular Subtypes (p

**Figure 3:**
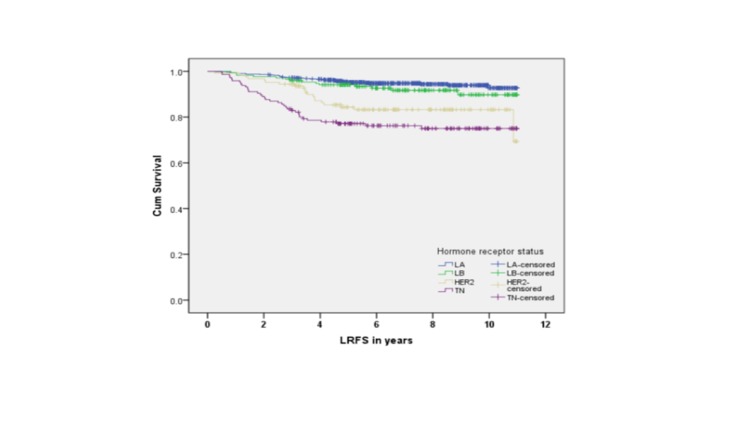
Local Recurrence-Free Survival by Subtypes (p LRFS = local recurrence-free survival

**Figure 4:**
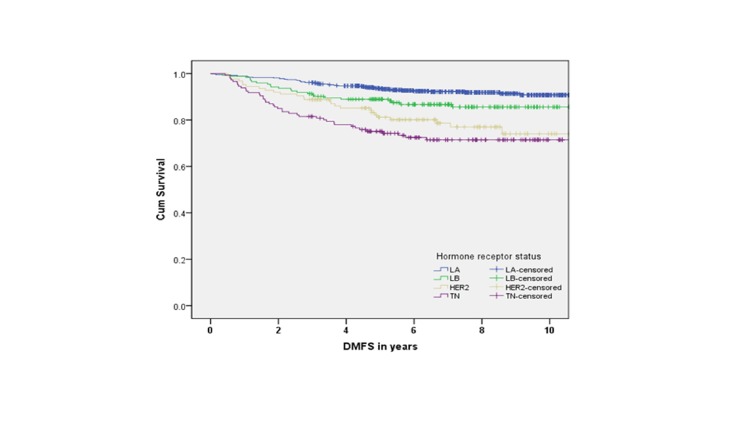
Distant Metastasis-Free Survival by Molecular Subtypes (p DMFS = distant metastasis-free survival

**Figure 5:**
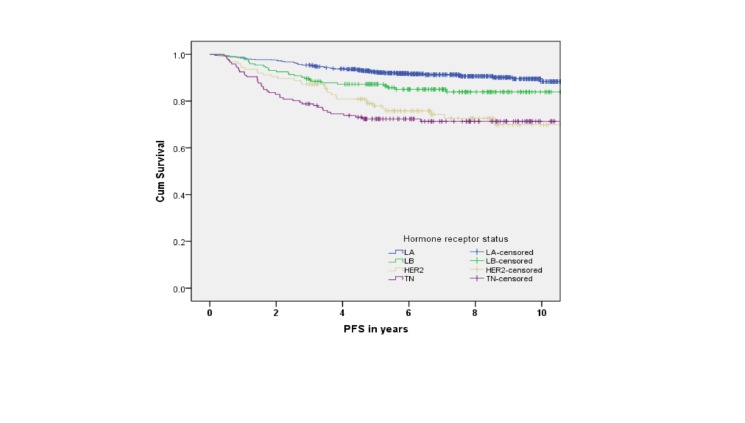
Progression-Free Survival by Molecular Subtypes (p PFS = progression-free survival

Five-year survival rates were 84% for LA, 83% for LB, 84% for TN, and 77% for HER2 subtypes. There was a statistically significant association between survival and molecular subtypes in an unadjusted analysis (Figures 2-5).

The medians for OS, PFS, LRFS, and DMFS were not reached. However, in all four endpoints, the survival was significantly shorter for TN compared to LA and LB subtypes (p < 0.001). Furthermore, the LA subtype had the best survival outcome in all endpoints (p < 0.001). In the adjusted multivariate analysis, the following variables were independent prognostic factors for survival: T stage, N stage, and molecular subtype (Table 3).

**Table 3:** Cox Regression Analysis for OS, LRFS, and DMFS OS: overall survival; LRFS: local recurrence-free survival; DMFS: distant metastasis-free survival; HR: hazard ratio

	OS				LRFS			DMFS		
Covariates	Hazard ratio	95% CI		P-value	HR	95% CI	P-value	HR	95% CI	P-value
TStage	1.41	1.04 - 1.91		0.027	1.154	0.86 - 1.54	0.327	1.808	1.43 - 2.29	< 0.001
N Stage	1.802		1.38 - 2.35	< 0.001	1.64	1.29 - 2.09	< 0.001	1.892	1.53 - 2.34	< 0.001
Grade	1.27		0.732 - 2.21	0.393	1.19	0.73 - 1.94	0.487	1.105	0.706 - 1.730	0.661
Molecular Type	1.737		1.39 - 2.17	< 0.001	1.66	1.36 - 2.02	< 0.001	1.644	1.371 - 1.973	< 0.001

Covariate	HR	95% CI	P value
Age (≤ 44 vs > 44)	0.809	0.44 - 1.49	0.498
Menopausal Status (pre vs post)	0.839	0.52 - 1.36	0.477
T-stage	1.94	1.53 - 2.46	< 0.001
N-stage	2.14	1.6 - 2.72	< 0.001
Tumor Grade	3.27	2.09 - 5.12	< 0.001
Histology	0.838	0.67 - 1.04	0.113
Chemotherapy	0.69	0.44 - 1.08	0.104
Boost	1.49	0.94 - 2.35	0.086
Molecular Subtypes	1.836	1.54 - 2.19	< 0.001

An increase of T Stage from 1 to 4 increases hazard rate six-fold and a change from N0 to N3 increases hazard ratio more than seven-fold. Hazard ratio (HR) increased by 40% for LB, 2.5-fold for HER2, and close to five-fold for TN subtypes when compared to LA.  

## Discussion

The identification of multiple molecular subtypes of breast cancer has allowed investigators to compare clinical BC outcomes amongst these subgroups. Many studies have demonstrated different recurrence and survival rates between subtypes [7-16]. In this retrospective review, we analyzed the post-radiotherapy pattern of failure and clinical outcome in a cohort of 1,088 patients. As expected, the most prevalent molecular subtype was LA, which is comparable to the Korean and Brazilian cohorts previously reported [8, 10]. Of the four subtypes, the LA subtype tends to have the best prognosis, fairly high survival, and low recurrent or metastases rates. The TN and HER2 subtypes of breast cancer were associated with a significantly poorer overall survival and prone to earlier recurrence and metastases. Our results demonstrate a significant association between molecular subtype and survival. The risk of death and relapse/metastases increases fewfold in TN compared to LA. Future prospective studies are warranted and could ultimately lead to the tailoring of adjuvant radiotherapy treatment fields based on both molecular subtype and the more conventional clinicopathologic characteristics. When we investigated these four breast cancer molecular subtypes with regard to patients’ age, the TN subtype tend to occur more often in younger women (< 45), which is consistent with the findings of Noh and Carey who reported the triple negative and HER2 subtype were more common in the young age group of African-American and Asian patients [9, 12]. 

The failure rate in our cohort was relatively low and comparable to 13.8% reported by Zhang, et al. [14]. Of the four subtypes, LA tends to have the lowest failure rate with low rates of local recurrence and distant metastasis. This could be due to fact that LA subtypes are usually ER+ and treatment for these tumors often includes hormone therapy [3, 17]. The TN and HER2 subtypes, on the contrary, were associated with higher rates of local recurrence and higher distant metastasis rates than LA subtypes, which is different from other studies showing that the TN and HER2 subtypes are not at significantly increased risk for local or local regional recurrence [8-9, 18-19] (Table 4). 

**Table 4:** Local Recurrence Difference in Molecular Subtypes HER2: human epidermal growth factor receptor-2; LA: luminal-A; LB: luminal-B; LR: local recurrence; SS: statistically significant; TN: triple negative; RR: recurrence rate

Author (Ref. #)	Publish Year	Number of patients	Median Follow-up (Years)	Local Recurrence Rate %	P - value
Haffty [7]	2006	482	7.9	TN: 17; Non-TN: 17	0.823
Dent [18]	2007	1601	8.1	TN: 13; Non-TN: 12	0.77
Freedman [19]	2009	753	5	TN: 3.2; Luminal: 2.3; HER2: 4.6	0.36
Ewan Millar [20]	2009	498	7	TN: 17.3; LA: 5.1; LB: 8.7; HER2: 15.4	0.012
Gabos [21]	2010	618	4.8	HER2: HR 11.13; 95% CI 2.78 - 44.53; TN: HR 4.72; 95% CI 1.53 - 14.52	SS
Kennecke [15]	2010	2,985	12	TN: 14; LA: 8; LB: 10; HER2: 21	0.005
Noh [9]	2011	596	6.6	Luminal: 4.1%, TN: 7.0%, HER2: 10.1%	0.151
Lowery [22]	2012	12,592	4.8	Luminal: RR 0.34; 95% CI 0.26 - 0.45); TN: RR 0.38; 95% CI 0.23 - 0.61); HER2: RR 1.44; 95% CI 1.06 - 1.95)	SS
Muanza [16]	2013	993	4.3	LA: 1.8; LB: 7.4; HER2: 6.8; TN: 11.4	0.001

For luminal B, our data is consistent with that reported by Tran, et al. with regards to the higher rate of local recurrence and distant relapse to bone and lung [13]. Breast or chest wall relapse was the most frequent site of local recurrence among LA - 61.5% (8/13), LB - 66.7% (4/6), and HER2 - 66.7% (2/3) groups. In contrast, regional lymph node relapse was the most common site of recurrence in 60.0% of TN cases (9/15). Bone was the most common site of DM in LA (41.7%), while LB and TN subtypes were most associated with lung metastasis (57.1% and 36.7%, respectively). The TN subtype was also associated with a relatively high proportion of brain metastasis at 33.3% (9/30). However, in the HER2 group, an even higher ratio of brain metastasis (50%) was observed and represented the most common site of DM in this group. This may be caused by trastuzumab’s inability to cross the blood-brain barrier and is compatible with Gabos’ report [21]. There was no statistical difference between sites of recurrence and metastasis, likely due to the relatively small number of events. However, it has been reported in other studies that LA and TN were respectively associated with bone metastasis and with visceral metastasis [19, 21]. To our knowledge, this study is the first to report that TN is associated with a higher incidence of regional lymph node recurrence. This could be of clinical significance and consideration whether to adjust radiation fields to cover regional lymph nodes is warranted, although other series have not shown a TN subtype association with higher regional lymph node involvement [20, 23-24]. Lowery’s recent review reports that patients with TN and HER2 breast tumors are at increased risk of developing LRR following breast-conserving therapy (BCT) or mastectomy [22]. Breast cancer subtypes should be taken into account when considering local control and potentially identify those at increased risk of LRR, who may benefit from a more aggressive local treatment. Another interesting finding in our series was that internal mammary lymph node involvement was also more commonly observed in the TN group - 33.3% (3/9). It begs the question as to whether we should consider internal mammary lymph node prophylactic irradiation in the TN subtype; however, additional prospective data is still needed to support this. The MA.20 randomized clinical trial demonstrated that adjuvant regional nodal irradiation reduces locoregional and distant recurrences and improves disease-free survival (DFS) with a trend to also improve OS in high-risk lymph node negative or node positive breast cancer treated with breast-conserving surgery and adjuvant systemic therapies [25]. The secondary analysis of this randomized trial database of more than 1,800 patients based on constructed molecular subtypes could provide additional information regarding the value of regional adjuvant radiotherapy for TN breast cancer patients. Although a boost to the surgical bed did not show a benefit with regard to overall survival for TN patients, our multivariate analysis revealed a benefit in local control (p = 0.049), similar to the one reported by Abdulkarim, et al. [26].  

We have demonstrated that the molecular subtype was the most significant factor associated with survival, with the TN subtype being the least favorable one. Braunstein, et al. reported similar results for disease-free survival in patients with locoregional recurrence after breast-conserving therapy [27]. In 82 patients with local recurrence, the risk of dying increased 4.5 folds in TN compared to the Luminal A subtype. Probable explanations for increased risk in TN subtype include ineffectiveness of hormonal therapy or HER2-directed agents.

The shortcomings of our research are that this is a retrospective study. In addition, the follow-up period of this cohort is relatively short with a low event rate. Our ongoing follow-up with the cohort would overcome this limitation and allow an examination of the long-term implications of different molecular subtypes on the survival and the pattern of recurrence of breast cancer patients.

## Conclusions

Of the four subtypes, the LA subtype tends to have the best prognosis, fairly high survival, and low recurrent or metastases rates. The TN and HER2 subtypes of breast cancer were associated with significantly poorer overall survival and were prone to earlier recurrence and metastases. Our results demonstrate a significant association between molecular subtype and survival. The risk of death and relapse/metastases increases fewfold in TN compared to LA. Future prospective studies are warranted and could ultimately lead to the tailoring of adjuvant radiotherapy treatment fields based on both the molecular subtype and the more conventional clinicopathologic characteristics.
